# Transient spontaneous chylothorax presented with neck pain and swelling in a patient with latent TB: Case report

**DOI:** 10.1002/ccr3.3550

**Published:** 2020-11-19

**Authors:** Hussam Almasri, Riyadh Hammamy, Ans Alamami, Mohamed Khalid Shariff

**Affiliations:** ^1^ Department of Medicine Hamad Medical Corporation Doha Qatar

**Keywords:** pleural effusion, spontaneous chylothorax, triglyceride

## Abstract

The aim of this case report is to raise awareness about self‐limited spontaneous chylothorax. Extensive workup for small nontraumatic chylothorax may not be justified.

## Graphical abstract

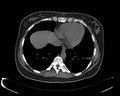

## 1 INTRODUCTION

Chylothorax is a relatively uncommon condition. It is characterized by the presence of chylous‐rich fluid within the pleural space. Spontaneous chylothorax usually refers to nontraumatic causes most commonly malignancy and infections. Previous reports showed patients with transient nontraumatic chylothorax with no identifiable cause and self‐limiting course.

We present a case of a middle‐aged woman found to have spontaneous chylothorax with evidence of resolution of pleural effusion without detectable cause.

Understanding the entity of transient spontaneous chylothorax is important to avoid invasive investigations in patient presenting with acute symptoms and no alarm signs.

Spontaneous chylothorax is a rare medical condition in which accumulation of chyle‐containing lymphatic fluid in the pleural cavity happens with no history of trauma. In general, chylothorax is associated with significant mortality and morbidity up to 10%.

Chylothorax is defined by the presence of high triglycerides and low cholesterol level in lymphatic exudative pleural effusion, most of the times the fluid appears milky on aspiration.^1^

The etiology of chylothorax varies from neoplasm to infections such as tuberculosis and many other causes.^9^

Transient spontaneous chylothorax was reported in few cases and was suggested to be due to minimal injury to the thoracic duct‐like sudden head movement and minimal exercise.^10^

Here, we present a case of young lady who suffered from neck pain and found to have transient chylothorax without history of trauma.

Our aim is to highlight the importance of this diagnosis and to avoid extensive testing in mild cases.

## 2 CASE PRESENTATION

A 35‐year‐old lady not known to have chronic medical illness. Presented to the hospital with chief complaint of left‐sided neck swelling and pain of 2 days duration. She did not have a history of fever, cough, weight loss, haemoptysis, chest pain, or shortness of breath. The patient reported no previous history of tuberculosis in the family or any sick contacts, and this was the first time to have such symptoms. The patient is a reformed smoker and drinks socially. There was no past history of trauma or any vigorous exercise.

Upon arrival to the emergency room, she was afebrile, blood pressure 104/64 mmgh, respiratory rate 18/minute, and pulse rate 83/minute.

Neck examination revealed left supraclavicular swelling, tender but soft with no palpable lymph nodes, examination of the chest revealed stony dullness, and reduced breath sounds in the basal left zone.

Ultrasonography revealed ill‐defined predominantly hyperechoic mixed echogenic area in the left supraclavicular region and left‐sided pleural effusion.

Computed tomography (CT) neck and chest revealed diffuse fat stranding and small lymph nodes noticed in mediastinum giving picture of mediastinitis/inflammatory process involving the left posterior neck muscle and in left pectoralis muscle with diffused smudged fat plane.

**FIGURE 1:**
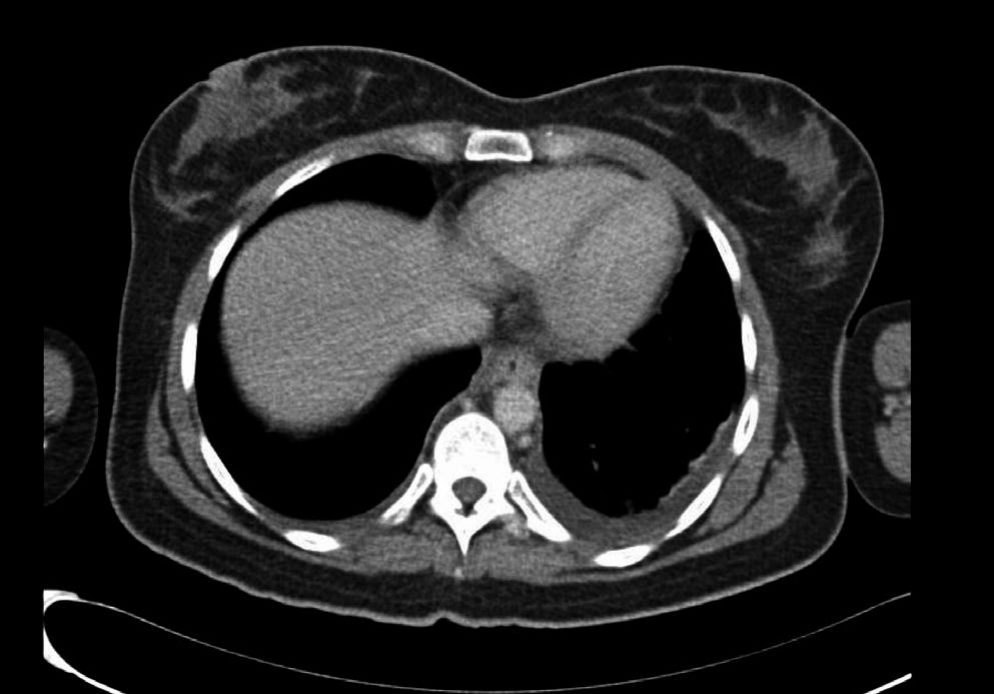
CT scan of the chest showing left‐sided pleural effusion with high attenuation suggestive of chylothorax

**FIGURE 2:**
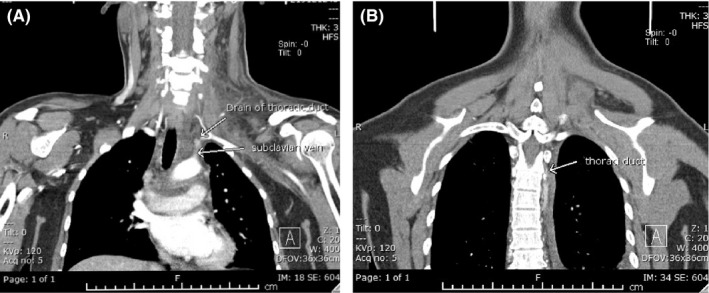
A and B, CT scan showing left neck subcutaneous edema, the track of thoracic duct

No collection noted in the neck. Mild left pleural effusion suggestive of chylothorax, Figure 1, Figure 2A,B.

Laboratory investigations including complete blood count, comprehensive metabolic profile and C‐reactive protein, lipase, and thyroid function tests were all within normal limits.

Serum triglyceride 1.9 mmol/L, normal limit 1.7‐5.6 mmol/L. Serum cholesterol 4.1 mmol/L, normal limit 5.2‐6.2 mmol/L.

Ultrasound‐guided diagnostic aspiration of the pleural fluid showed milky alkalotic exudative fluid with predominant lymphocytes, triglycerides level of 2.39 mmol/l, cholesterol level of 3.4 mmol/l, negative gram stain and bacterial culture, negative acid‐fast bacilli smear, culture and TB‐PCR, and also negative cytology. QuantiFERON was positive.

The patient's symptoms started to improve during hospital stay with symptomatic treatment.

**FIGURE 3:**
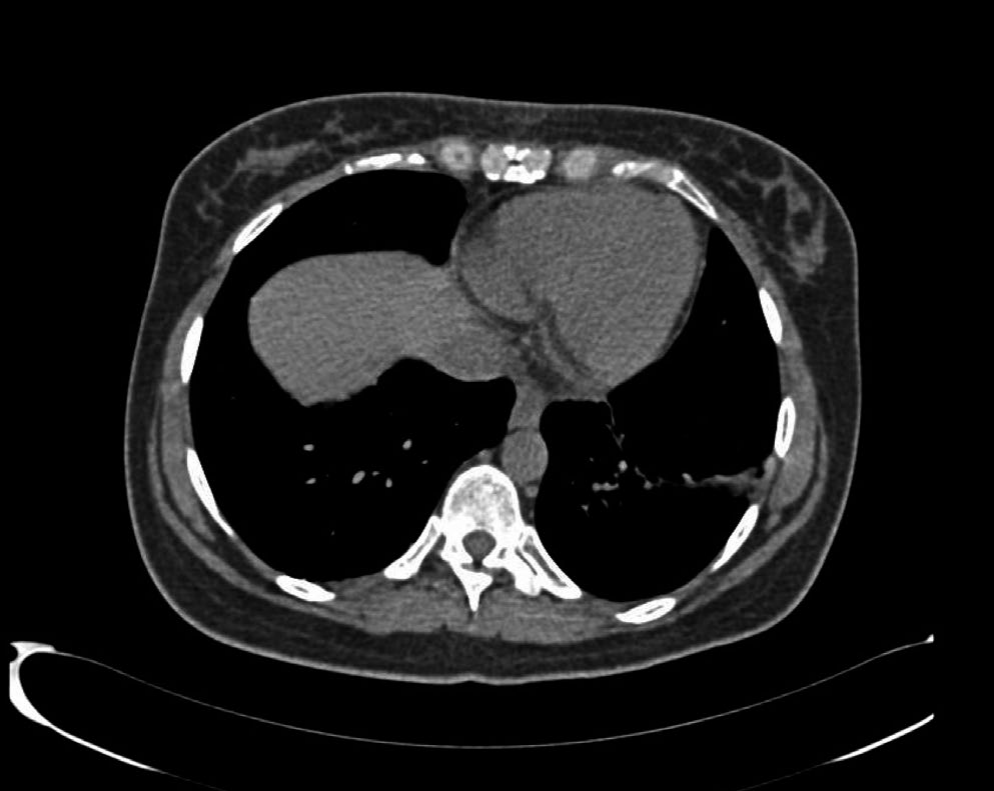
Follow‐up CT scan showed resolution of the left‐sided pleural effusion

Few days later, the patient underwent repeated CT scan which showed resolution of most of the pleural effusion with normal abdomen CT findings (Figure 3).

Follow‐up chest X‐ray after 1 month was unremarkable, and the patient was free of symptoms.

## 3 DISCUSSION

Chylomicrons arise from the combination of long‐chain triglycerides with cholesterol esters and phospholipids. These molecules are resistant to be broken down by intestinal lipase so they pass to the lymphatic system of the small intestines then via thoracic duct to systemic circulation.^1^

The thoracic duct length varies from 38 to 45 cm and ends typically at the junction between internal jugular and left subclavian veins carrying lymph from lower limbs and chyle from intestines.^10^

Chylothorax results from the leak of chyle from thoracic duct to the pleural cavity. This can happen after rupture, disruption, or obstruction of the thoracic duct.^1^, ^6^

The etiology of chylothorax can be categorized into traumatic, nontraumatic (spontaneous), and idiopathic. Chylothorax usually present with acute symptoms including dyspnoea, fatigue, and less commonly chest pain and fever. Chyle is a nonirritating fluid to the pleura, and this can explain the infrequency of chest pain upon presentation.

The diagnosis of chylothorax is mainly based on aspirated fluid analysis, the milky appearance is not exclusive to chylothorax, other conditions such as empyema and cholesterol effusion can cause milky pleural effusion and the differentiation between these conditions is vital for management.^8^, ^10^

Chylothorax is defined by the presence of triglycerides more than 110 mg/dL in the fluid and cholesterol less than serum. Usually, the fluid is alkalotic, lymphocytic, and exudative,^8^, ^10^ but it was reported to be transudative rarely.^15^

Previously, nontraumatic chylothorax especially from neoplasms was the most common in adults but recent reports indicated that traumatic mainly postoperative cases are more common, this can be due to increased number of chest procedures and surgery or only increased recognition.^1^, ^4^

Traumatic chylothorax was described in various types of surgery including chest, neck, cardiac, gastric, and esophageal. It is considered to be iatrogenic with favorable outcomes in most cases. Esophageal surgery was considered the highest risk surgery to develop chylothorax. In addition to surgery, blunt trauma and penetrating injuries were also associated with chylothorax due to direct or indirect thoracic duct damage.^1^, ^4^

Most cases of nontraumatic or spontaneous chylothorax come from malignancy with lymphoma to be most common followed by bronchogenic carcinoma and other tumors, kaposi sarcoma was also reported.^4^

Other than neoplasms, many causes were identified to result in chylothorax including tuberculosis, filariasis, sarcoidosis, congestive heart failure, yellow nail syndrome, lymphangioleiomyomatosis, lymphatic malformation, and radiation therapy.^2^, ^3^, ^4^, ^12^

Congenital chylothorax was described in newborns to be the most common cause. Trisomy 21 or Turner syndrome appears to be associated with risk factor.

Spontaneous chylothorax with no apparent cause was reported in few cases, minimal physical activity or sudden head movement especially neck hyperextension was thought to be the precipitating factor along with recurrent vomiting, hiccups, and cough.^9^, ^11^ It was suggested that there should be a weak point from pre‐existing disease to cause thoracic duct rupture with minimal exercise, reported cases had either active or previous TB infection,^9^, ^13^ the presence of positive QuantiFERON raise the possibility of latent TB which could have played a role in causing disruption of the thoracic duct. There was no evidence of active TB in our patient, and the spontaneous resolution of pleural fluid can support that.

Management of Chylothorax can be either conservative or surgical. Conservative treatment includes the use of a low‐fat diet supplemented with medium‐chain triglycerides (MCT), and other interventions are available based on the case including chest tube drainage, pleurodesis, thoracic duct ligation, or embolization.^5^, ^7^, ^14^

But its worthwhile to know that most of the cases of spontaneous chylothorax are self‐limiting and can be managed conservatively with rest, good hydration, and low‐fat diet.

## 4 CONCLUSIONS

Spontaneous chylothorax can be the result of variety of conditions. It should be always in mind that some cases are transient and no underlying disorder can be identified. We recommend higher threshold for invasive investigations when no alarm signs are detected.

## 5 PATIENT PERSPECTIVE

“It was the first time having these symptoms. I was relieved that the illness is benign and will not need more procedures.”

## CONFLICT OF INTEREST

All authors declare no conflict of interest.

## AUTHOR CONTRIBUTIONS

HA: involved in manuscript writing, literature review, correspondence. RH: involved in literature review and manuscript writing. AA: involved in literature review. MKS: involved in clinical follow‐up.

## ETHICAL APPROVAL

Medical Research Center, Hamad Medical Corporation, MRC‐04‐20‐628. Patient consent has been obtained for publication of case details.
